# The Training of Hospital Matrons

**Published:** 1916-11-25

**Authors:** 


### THE MATRONS' AND SISTERS' DEPARTMENT.

### THE TRAINING OF HOSPITAL MATRONS.

By ONE OF THEM.

WHAT qualities should the ideal matron possess? is a question of perennial interest. It will probably be generally agreed that she should be just, far-seeing, a good administrator, accurate in judgment, of a calm, equable disposition, educated, courteous, sympathetic, a good teacher and lecturer, a trained nurse of experience, and worldly in the best sense of the word; *i.e.*, able to take an interest in many matters and to know how to deal with persons of all classes. How nearly does the average matron approach to this ideal? If she has not these qualities in a marked degree before training, has her training tended to give them to her? If she originally had them, has the training developed her natural powers, or has it made her narrow-minded, autocratic, and inclined to be bound by her own walls?

Matrons are usually hard-working, conscientious workers, but those of wide interests, even of wide nursing interests, are few and far to seek. Many are so absorbed with their work that they seldom try to come into contact with their equals in their profession, let alone with women working in other professions, and are content to be little tin gods in their own building. A large number of matrons do not take a daily paper; the majority probably take one or perhaps two nursing papers, but often find no time to read them. Those who have been through the training, held administrative posts, and then taken up another profession, or for some reason ceased to hold posts in the nursing world, and have time to look back, can see what a narrowing effect hospital training has. The three years seem to be made up of long hours of work, great fatigue, constant studying for examinations, anxiety about ward work, then about ward management, with days or half-days off duty; no time for reading, study (other than for nursing examinations), recreation, continuing a hobby or retaining an accomplishment.

Then when the three years are over, and the magic piece of parchment is in the owner's hands, is her training complete? No; she then has to decide what branch of nursing she will take up. If she decides that she will do hospital administrative work, where and how can she get the necessary training? Our ideal matron should have a knowledge of private and district nursing, certainly the latter; she should have been a ward sister, home sister, under a trained housekeeper, and an assistant matron; she should have her midwifery certificate, and at all events should know enough massage to be able to supervise the massage department in a hospital. Under what conditions does she acquire all this experience? Has she any more leisure than she had during her three years' training, or energy to enable her to have any real recreations beyond possibly a theatre occasionally, or a long 'bus ride!

What time during all these five or six years has the trained head for any self-development, meeting and making new friends, besides actual hospital ones? Can one wonder that the matron is often narrow-minded, knows little of outside interests, is at times autocratic? She seldom or never meets her equals or superiors in the nursing world. She thinks that the system which sufficed for training in her day and in her training school is the one and the only one to-day. Seldom meeting women of other professions, she does not realise the advance that has been made regarding women's work, and that unless the matrons of to-day bestir themselves and make the training a more suitable one for the educated girl of the present time, nursing in the future must fall back to the Sarah Gamp state. Matrons should try to arrange the hours on duty for their staff so that they have more time for real recreation. They should meet and try to form some scheme by which the matrons of the future may receive definite training, preferably on the College or University system, to fit them for their responsible work.

